# Appraisals and coping mediate the relationship between resilience and distress among significant others of persons with spinal cord injury or acquired brain injury: a cross-sectional study

**DOI:** 10.1186/s40359-020-00419-z

**Published:** 2020-05-20

**Authors:** Eline W. M. Scholten, Julia D. H. P. Simon, Tijn van Diemen, Chantal F. Hillebregt, Marjolijn Ketelaar, Kees Hein Woldendorp, Rutger Osterthun, Johanna M. A. Visser-Meily, POWER Group, C. C. M. van Laake - Geelen, J. Stolwijk, C. A. Dijkstra, E. Agterhof, D. Gobets, E. M. Maas, H. van der Werf, C. E. de Boer, M. Beurskens, I. van Nes, T. van Diemen, K. H. Woldendorp, J. Hurkmans, M. Luijkx, D. C. M. Spijkerman, R. Osterthun, J. Sprik-Bakker, E. Roels, M. Hoonhorst, Marcel W. M. Post

**Affiliations:** 1 Center of Excellence for Rehabilitation Medicine, UMC Utrecht Brain Center, University Medical Center Utrecht, and De Hoogstraat Rehabilitation, Utrecht, the Netherlands; 2 Sint Maartenskliniek, Department of Rehabilitation, Nijmegen, the Netherlands; 3 University of Groningen, University Medical Center Groningen, Center for Rehabilitation, Department of Rehabilitation Medicine, Groningen, the Netherlands; 4 “Revalidatie Friesland” Center for Rehabilitation, Beetsterzwaag, the Netherlands; 5 Department of Rehabilitation Medicine, Erasmus MC, Rotterdam, the Netherlands; 6 Rijndam Rehabilitation Center, Rotterdam, the Netherlands; 7 Department of Rehabilitation, Physical Therapy Science & Sports, UMC Utrecht Brain Center, University Medical Center Utrecht, Utrecht, the Netherlands

**Keywords:** Psychological stress, Spinal cord injuries, Brain injuries, Psychological adaptation, Cognitive processes, Caregiver

## Abstract

**Background:**

Many significant others of persons with serious conditions like spinal cord injury (SCI) and acquired brain injury (ABI) report high levels of psychological distress. In line with the stress-coping model, the aim of the present study was to investigate the relationship between personal resource resilience and psychological distress, and whether appraisals of threat and loss, and passive coping mediate this relationship.

**Methods:**

Significant others (*n* = 228) of persons with SCI or ABI completed questionnaires shortly after admission to first inpatient rehabilitation after onset of the condition. The questionnaire included measures to assess psychological distress (Hospital Anxiety and Depression Scale), resilience (Connor-Davidson Resilience Scale-10), appraisals (Appraisals of Life Events scale, threat and loss) and passive coping (Utrecht Coping List). The PROCESS tool was used to test the presence of mediation. Confounding and differences between SCI and ABI were investigated.

**Results:**

High levels of psychological distress among significant others were found (34–41%). Fifty-five percent of the variance in psychological distress was explained by the relationship between resilience and psychological distress. This relationship was mediated by appraisals of threat and loss, and passive coping. The relationship between resilience and psychological distress was similar in the SCI and ABI groups.

**Conclusions:**

The results of our study indicate that appraisals of threat and loss and passive coping are mediating factors in the relationship between resilience and psychological distress. It seems useful to investigate if interventions focussing on psychological factors like resilience, appraisal and coping are effective to prevent or reduce psychological distress among significant others of persons with SCI or ABI.

**Trial registration:**

Dutch trial register NTR5742. Registered January 9, 2016.

## Background

Spinal cord injury (SCI) and acquired brain injury (ABI) are two major causes of chronic disability worldwide [1]. Both conditions often have long-term effects that impact the lives of the persons themselves, but also that of the persons close to them, their significant others [2–4]. Although the new situation may have some positive aspects for significant others (e.g. self-esteem derived from caregiving) [5], they often report high levels of psychological distress in terms of anxiety and depression, and these levels of psychological distress remain high on the long term [6, 7]. To be able to support significant others with substantiated interventions to treat psychological distress, it is important to understand the mechanism underlying caregiving-related psychological distress. There is a very long history of stress-response theory which has resulted in numerous theoretical models explaining well-being outcomes [8, 9]. The stress-coping model, originally proposed by Lazarus and Folkman, is a widely recognized theoretical model often used to explain psychological distress and has been primarily used to explain emotional well-being among persons with SCI [10, 11].

### Stress-coping model

According to the model, in situations of stress, a person’s health-related quality of life (e.g. emotional well-being) is the outcome of the interplay between several factors. The trigger in this interplay is the stressful situation. How the stressful situation is evaluated depends on the person’s own personal resources, health-related factors and the social and physical context. The cognitive process of evaluation is called appraisal. Coping refers to how persons tend to react, based on this appraisal, to solve personal and interpersonal problems in order to try to master, minimize or tolerate stress and conflict [10]. How the person copes with the stressful situation affects the adjustment outcomes.

### The stress-coping model to explain psychological distress among significant others

Being a significant other of a person with SCI or ABI can be considered as a stressful situation [4]. This suggest the possible applicability of the stress-coping model in the explanation of adjustment outcomes among significant others. The application of the model can provide theoretical based insight which is important to be able to substantiate the support for significant others. However, there is still little evidence which support the applicability of the model on significant others of persons with SCI or ABI. Some evidence is found in research conducted in other diagnosis groups. Among caregivers of patients with prostate cancer was found that personal resources (including self-efficacy) were longitudinally associated with quality of life, and that this relationship was partly mediated by negative appraisals and avoidant coping [12]. Among caregivers of individuals with traumatic brain injury some support for the stress-coping model was found in a study using regression analysis to predict quality of life, which had demonstrated that appraisal was a strong predictor [13]. However, in this study the association between coping and quality of life disappeared after controlling for other variables, and the mediating effect of appraisal and coping in the explanation of quality of life was not tested.

Indications for the applicability of the stress-coping model to explain psychological distress among significant others are predominantly found in bivariate relationships between separate elements of the model. First, resilience – which reflects one’s ability to thrive in the face of adversity – seems to be an important expression of personal resource [14, 15]. Previous research among significant others of persons with SCI or cancer showed that resilience is a strong predictor of psychological distress [16–18]. Furthermore, negative appraisals and passive coping strategies were found to be correlated with higher levels of psychological distress among significant others with stroke [19–21]. If appraisals and coping mediate the relationship between resilience and psychological distress, as can be expected based on the stress-coping model, is still unclear.

### Present study

Based on the stress-coping model, the objective of this study is to test if psychological distress – as indicator of emotional well-being outcomes – among significant others can be explained by the personal resource resilience, and if this relation is serially mediated by appraisals and coping. This study targeted significant others of persons with SCI or ABI in the subacute phase during first inpatient rehabilitation. We focus on SCI and ABI because these are two major causes of chronic disability which differ in presence and consequences [1]. Therefore, we will also investigate the relationships in both subgroups separately.

## Methods

### Design

We used baseline data of the cohort part of the POWER-study [22]. The aim of this cohort study is to identify predictors at admission to inpatient rehabilitation of long-term empowerment problems among persons with SCI or ABI and their significant others. Recruitment took place between April 2016 and July 2018. The Medical Ethics Committee of the University Medical Center Utrecht declared that this study did not need approval according to the Dutch Law on Medical Research (protocol number 15–617/C). Permission to execute the study was granted by the boards of all twelve participating Dutch rehabilitation centers. We certify that we followed all applicable institutional and governmental regulations concerning the ethical use of human volunteers during the course of this research.

### Participants

In the POWER-study couples of persons with SCI or ABI and their significant others were included [22]. Inclusion criteria for the person with SCI of ABI were: first inpatient rehabilitation after onset of injury and expected stay in the rehabilitation center for at least 4 weeks. Because POWER was designed to investigate the long-term impact of chronic injuries, persons with SCI or ABI were excluded when (nearly) full recovery was expected, no return to home was expected, or if they had a limited life expectancy. Persons with severe cognitive or intellectual problems were excluded due to their inability to complete the questionnaires. Cognitive or intellectual problems were defined as restrictions in the expression and/or understanding of language and were assessed by nurses based on their clinical view and the Dutch aphasia scale [23]. Persons with SCI or ABI named their significant other, usually their partner, but it could also be a child, parent, sibling, other family member, or friend. Persons were excluded if they could not name a significant other or if this significant other declined participation. All participants had to be ≥18 years of age. The present study focused exclusively on significant others.

### Procedure

Shortly after admission of the person with SCI or ABI to one of the participating rehabilitation centers and after signing informed consent, significant others were asked to complete a self-report questionnaire (print or electronically). Diagnosis-specific information of the person with SCI or ABI was extracted from the patient’s file.

### Measures

#### Dependent variable

Psychological distress was measured with the Hospital Anxiety and Depression Scale (HADS) [24, 25], which is considered an effective measure of general psychological distress [26]. The HADS consists of 14 items reflecting symptoms of anxiety and depression by seven items each. Every item is scored on a four-point scale, ranging 0 (“no symptoms”) to 3 (“maximum impairment”). A total sum score was calculated (range 0–42), where higher scores indicate higher psychological distress. Cut-off scores for the HADS focus on sum scores of anxiety and depression subscales separately (range 0–21), where scores of ≥8 indicate high anxiety or depressive symptoms [27]. The HADS has shown good psychometric properties in different populations [25]. Cronbach’s alpha of the total scale was .91 in the current study.

#### Independent variable

Resilience was measured with the ten-item version of the Connor-Davidson Resilience Scale (CD-RISC-10) [14, 28]. Participants rated ten statements on a five-point scale ranging 0 (“not true at all”) to 4 (“true nearly all the time”). Total scores range between 0 and 40, where higher scores indicate higher resilience capacity. The CD-RISC-10 has shown good internal consistency and construct validity [28]. In the current study, we found a Cronbach’s alpha of .92.

#### Mediators

Appraisal is the first potential mediator. Three common appraisal patterns have been identified in response to stressful situations: appraisals of threat (potential for harm), loss (potential for disintegration of friendships, health, or self-esteem), and challenge (potential for growth, gain, and mastery) [29]. Previous research showed that in particular negative appraisals predict greater negative outcomes, e.g. anxiety [20]. Based on that, in the design of the study we have decided only to assess appraisals of threat and loss, and not appraisals of challenge. In addition, we found it undesirable to confront significant others of persons recently confronted with SCI or ABI with questions such as: “I find my current circumstances enjoyable”. So, we decided to focus on appraisals of threat and loss. Appraisals were measured with the threat (6 items) and loss (4 items) subscales of the Appraisals of Life Events (ALE) scale [29]. Participants rated the extent to which different adjectives describe their perceptions of their current life circumstances (0 = “not at all” to 5 = “very much so”). Subscale scores were computed as the mean item scores in that subscale. For this study a total score was computed as the mean of the two subscale scores (range 0–5) so that both subscales contributed equally to the total score. Higher scores indicate higher appraisals of threat and loss. The complete ALE has shown good psychometric properties [29]. Cronbach’s alpha was .93 in the current study.

Coping is the second potential mediator. Previous research has shown that a passive coping strategy was most strongly associated with negative psychological outcomes [12, 19]. Therefore, we decided to focus on passive coping which was operationalized as the tendency of being completely absorbed by and unable to deal with a stressful situation, retreating into oneself, and worrying about the past [30]. Passive coping was measured with the passive reaction pattern subscale of the Utrecht Coping List (UCL) [30, 31]. This subscale consists of seven items, scored on a four-point scale ranging from 1 (“rarely true”) to 4 (“true nearly all the time”). The total sum score ranged from 7 to 28, where higher scores indicated a greater tendency to adopt a passive coping style. The UCL has shown good reliability and validity [32]. Cronbach’s alpha was .75 in the current study.

#### Potential confounders

Demographic information included: sex (male = 0, female = 1), age (years), nationality (Dutch = 0, non-Dutch = 1), higher education (i.e. finished bachelor degree or higher) (no = 0, yes = 1), and relationship with the person with SCI or ABI (0 = no partner (e.g. child, parent, sibling or friend), 1 = partner). Health-related factors included diagnosis (SCI = 0, ABI = 1) and cause of injury (0 = traumatic, 1 = non-traumatic). For SCI, the American Spinal Injury Association (ASIA) Impairment Scale (AIS) score was determined by a trained rehabilitation physician [33]. The AIS provides information about sensory/motor completeness of the SCI. Furthermore, the level of injury (paraplegia (0) or tetraplegia (1) was assessed, where paraplegia was defined as a lesion at or below the first thoracic segment, tetraplegia as a lesion at or above the first thoracic segment [34]. For ABI, location of injury was specified in left hemisphere, right hemisphere, both hemispheres, or brainstem. Physical independence for both diagnosis groups was measured with the physical independence subscale of the Utrecht Scale for Evaluation of Rehabilitation (USER) [35]. This subscale consists of 14 items on independence in mobility and self-care which are scored on a six-point scale (range 0–5) by an involved professional. The total score ranged from 0 to 70. A higher score represents better physical independence [36]. The USER is a valid and responsive scale [36]. Total USER scores were extracted from patients’ files. We did not have the USER data at item level, therefore we were not able to calculate the Cronbach’s alpha based on our own data. In a former Dutch study, Cronbach’s alphas showed satisfactory internal consistency (0.89–0.90) [35].

### Statistical analyses

**Fig. 1:**
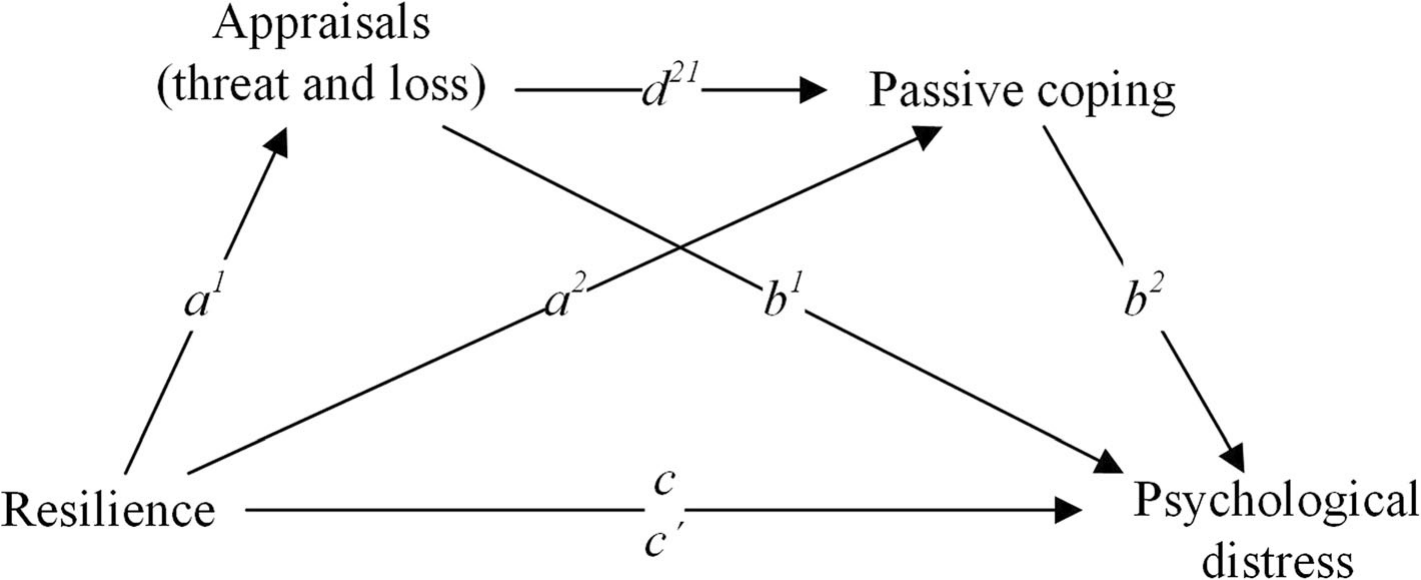
Serial multiple mediation model. Adapted from Hayes AF. Multiple mediator models. In: Hayes AF, editor. Introduction to mediation, moderation, and conditional process analysis: A regression-based approach. New York, USA: Guilford Publications; 2013. p. 446

We used descriptive statistics to describe the study population and outcome variables. Differences between SCI and ABI groups were tested with independent samples t-tests and Pearson’s rho correlations were computed to assess the relationships between the dependent, independent, (possible) mediating variables and the potential confounders. The stress-coping model assumes serial mediation. However, it is difficult to test serial mediation with standard linear regression. Therefore, as an application of regression, we used the PROCESS tool which provides a serial multiple mediation model that can be used to investigate the direct relationship between a predictor (resilience) and outcome (psychological distress) as well as indirect relationships via one or more mediators (appraisals of threat and loss, and passive coping) [37]. Unstandardized regression coefficients were calculated for each path in the mediation model, displayed in Fig. 1. The total effect of resilience on psychological distress without mediating variables is represented in *c*, and *c’* represents the direct effect of resilience on psychological distress while partialling out the effects of both mediators (appraisals of threat and loss, and passive coping). The indirect effect of resilience on psychological distress is calculated as the sum of the effects of different pathways including mediators: effect of resilience on psychological distress via appraisals only, via coping only, and via appraisals and coping. The effects are calculated by multiplying the coefficients of the pathways, so, the pathway via appraisals only is calculated by multiplying *a*^*1*^ and *b*^*1*^. Of the indirect effects, the bias-corrected 95% confidence intervals were based on 10.000 bootstrapped resamples [37, 38]. When zero is not included in a bias-corrected 95% confidence interval, it can be concluded that in 95% of the bootstrapped samples the effect is significant.

Possible confounders, namely demographic (sex, age, nationality, education, and relationship with the person with SCI or ABI) and health-related factors (diagnosis and physical indepencence) were added as covariates in de mediation model if they were significantly correlated with the main outcome variable psychological distress and the predictor (resilience) or mediator(s) (appraisals of threat and loss and/or passive coping). Afterwards, the serial multiple mediation model was tested separately for the SCI and ABI groups to explore the differences between these groups.

We analysed the data with IBM SPSS Statistics 25. The internal consistencies of the used scales were assessed by calculating the Cronbach’s alphas. Although the alpha of the UCL was somewhat lower than the alphas of the other scales, all scales had a value of ≥0.7 and, therefore, were interpreted as satisfactory [39]. A significance level of *p* < .05 (two-tailed) was used. We used Cohen’s standards to interpret the correlations (*r* = .10, weak; *r* = .30, moderate; and *r* = .50, strong) [40]. Individuals with missing scale scores were excluded from further analyses. We have checked the regular assumptions for multiple regression analysis: normality of the error terms (normal probability plot), linearity and homoscedasticity (plot of the residuals versus the predicted values of the dependent variable), independence of the error terms (Durbin Watson statistic) and collinearity (values of the variance inflation factors) [41, 42]. We found no indications of violation of one of the assumptions.

## Results

### Sample characteristics

Data of 237 significant others were available, of which nine were excluded because of missing scores on the HADS (4 cases), CD-RISC-10 (3 cases) or ALE (2 cases), resulting in a sample of 228 caregivers. The median number of weeks between onset of injury and completing the questionnaire was 5 weeks and did not differ between diagnoses. Table 1 displays characteristics of the caregivers and persons with SCI or ABI. The most common traumatic cause of SCI was a fall (17.2% of all persons with SCI), and the most common non-traumatic cause was spinal degeneration (18.9%). The large majority of ABI were of non-traumatic origin, and in about half of all persons with ABI, the cause was a cerebral infarction (54.3%). Most significant others were partner (72.4%), others were child (13.2%), parent (8.8%), other family member (3.1%), friend (2.2%) or neighbor (0.4%).

**Table 1:** Characteristics of significant others and persons with disabilities

	**Total (*n*** **= 228)**		**SCI (*n*** **= 122)**		**ABI (*n*** **= 106)**	
***Significant others***	***n***	***n*(%) / mean (SD), range**	***n***	***n*(%) / mean (SD), range**	***n***	***n*(%) / mean (SD), range**
Sex (female)	228	149 (65.4)	122	92 (75.4)*	106	57 (53.8)*
Age in years	224	54.1 (12.8), 23–82	120	54.4 (13.7), 25–82	104	53.9 (11.8), 23–75
Nationality (non-Dutch)	226	17 (7.5)	121	9 (7.4)	105	8 (7.5)
Education level (high)	223	86 (38.6)	118	41 (34.7)	105	45 (42.9)
Relationship with person with SCI/ABI (partner)	228	165 (72.4)	122	86 (70.5)	106	79 (74.5)
***Injury information***						
Physical independence	218	34.6 (18.8), 0–70	120	27.7 (17.4), 0–70*	98	43.0 (17.0), 5–70*
Cause (non-traumatic)	227	154 (67.8)	122	65 (53.3)*	105	89 (84.8)*
AIS^a^ (SCI only)	─	─	121		─	─
	─	─	A	15 (12.4)	─	─
	─	─	B	18 (14.9)	─	─
	─	─	C	23 (19.0)	─	─
	─	─	D	65 (53.7)	─	─
Level/location injury	─	─	122		99	
	─	─	Paraplegia	59 (48.4)	Left	41 (41.4)
	─	─	Tetraplegia	63 (51.6)	Right	35 (35.3)
	─	─	─	─	Both sides	18 (18.2)
	─	─	─	─	Brainstem	5 (5.1)

*Note. SCI* Spinal cord injury, *ABI* Acquired brain injury, *SD* Standard deviation, *n* Number of participants

*Independent samples t-test showed a difference in sex (*t* (209.0) = 3.5, *p* < .01), physical independence (*t* (209.2) = − 6.5, *p* < .001) and cause of injury (*t* (215.5) = 5.3, *p* < .001) between SCI and ABI. ^a^AIS = American Spinal Injury Association Impairment Scale. A = complete; B = sensory incomplete; C = motor incomplete with less than half of key muscle functions below the single neurological level of injury having a muscle grade ≥ 3; D = motor incomplete with at least half of key muscle functions below the single neurological level of injury having a muscle grade ≥ 3 [33]

### Psychological distress

Mean variable scores, standard deviations, and independent samples t-tests between SCI and ABI groups are shown in Table 2. For the anxiety and depression subscales of the HADS respectively, 40.8 and 33.8% of the total group of significant others had a score of ≥8, indicating high anxiety or depressive symptoms (in the SCI group respectively 45.9 and 39.3%; in the ABI group 34.9 and 27.4%). Significant others of persons with ABI showed to be more resilient and had fewer appraisals of threat and loss compared with significant others of persons with SCI.

**Table 2:** Means, standard deviations and independent samples t-tests between SCI and ABI groups (*n* = 228)

	**Total**		**SCI**		**ABI**		**Independent samples t-test**^**a**^		
	**(*n*** **= 228)**		**(*n*** **= 122)**		**(*n*** **= 106)**				
**Variable (range of scores)**	**M**	**SD**	**M**	**SD**	**M**	**SD**	***t***	***df***	***p***
1. Resilience (0–40)	28.3	5.9	27.5	6.0	29.1	5.8	−2.02	226	<.05
2. Appraisals (threat and loss) (0–5)	1.3	1.1	1.6	1.2	1.0	1.0	4.01	225.6	<.001
3. Passive coping (7–28)	10.5	2.8	10.8	2.9	10.2	2.6	1.61	226	.11
4. Psychological distress (0–42)	13.1	7.8	14.0	7.6	12.0	7.8	1.95	226	.05

*Note. SCI* Spinal cord injury, *ABI* Acquired brain injury, *M* Mean, *SD* Standard deviation, *n* Number of participants, *t* t-value, *df* Degrees of freedom, *p p*-value

^a^Independent samples *t*-test to test differences in scale scores between SCI and ABI

### Correlations and mediation model

Correlations between resilience, appraisals of threat and loss, passive coping, and psychological distress were all moderate to strong (.40–.67), and in the expected direction based on the stress-coping theory (see Table 3). None of the potential confounders was significantly related with the dependent variable psychological distress and the predictor (resilience) or one of the mediators, and therefore no covariates were added in the serial multiple mediation model.

**Table 3:** Pearson’s r correlation coefficients of the study variables (*n* = 228)

Variable	**1.**	**2.**	**3.**	**4.**
1. Resilience	–	–	–	–
2. Appraisals (threat and loss)	−.40***	–	–	–
3. Passive coping	−.44***	.54***	–	–
4. Psychological distress	−.42***	.67***	.61***	–
5. Sex (female)	−.06	.05	.05	.02
6. Age	−.01	.01	−.19**	.03
7. Nationality (non-Dutch)	.09	−.07	−.02	−.04
8. Education (high)	.14*	−.08	−.05	−.11
9. Relationship with person with SCI/ABI (partner)	.09	.07	−.09	.09
10. Diagnosis (ABI)	.13*	−.23***	−.11	−.13
11. Physical independence (person with SCI/ABI)	.04	−.12	.04	−.04

*Note.* SCI = spinal cord injury; ABI = acquired brain injury; *n* = number of participants

*p < .05; **p < .01; ***p < .001

**Fig. 2:**
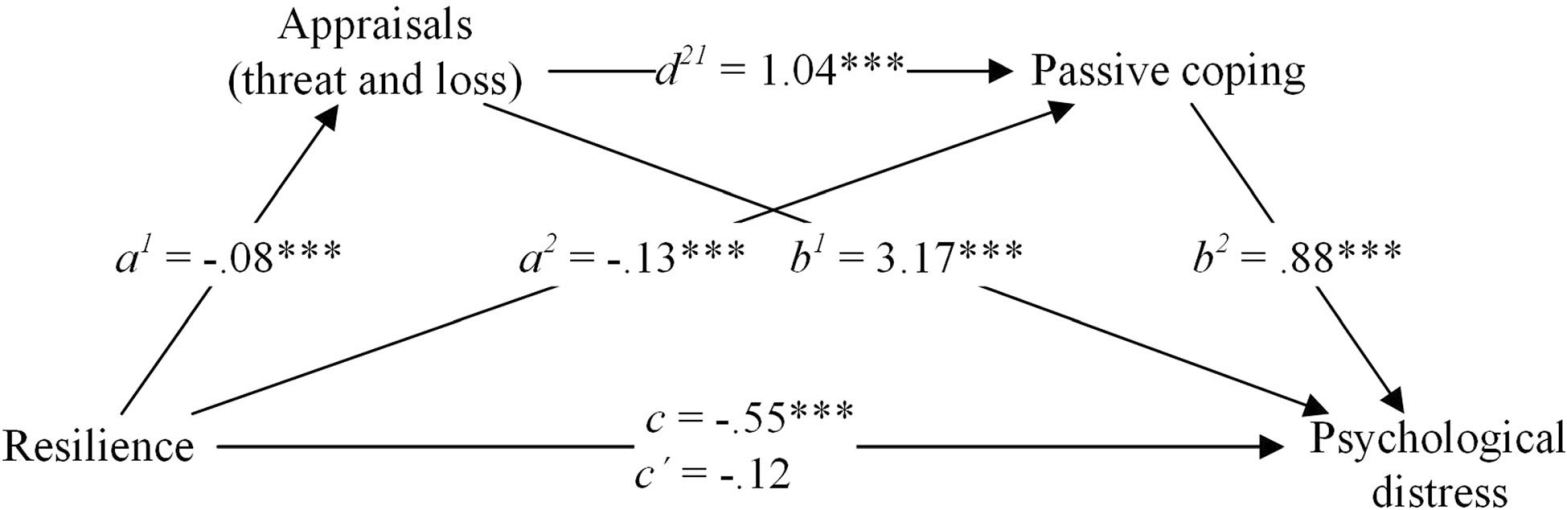
Serial multiple mediation model (with coefficients)

Table 4 and Fig. 2 show the results of the mediation analysis. The complete model explained 55% of the variance in psychological distress. Without the mediators, the regression coefficient between resilience and psychological distress was −.55 (*p* < .001) (Fig. 2 and Table 5). After adding the mediators to the model, this coefficient decreased and was no longer statistically significant (*c’* = −.12, *p* > .05). The original relationship between resilience and psychological distress was explained by indirect pathways, mostly by the indirect relationship via appraisals of threat and loss only (*a*^*1*^ * *b*^*1*^ = −.24), followed by the indirect relationship via passive coping only (*a*^2^* b^*2*^ = −.11), and the indirect relationship via appraisals of threat and loss, and passive coping (*a*^1^* d^21^* b^*2*^ = −.07).

**Table 4:** Regression coefficients, standard errors, and model summary information for the presumed serial multiple mediator model (*n* = 228)

	**Appraisals of threat and loss**				**Passive coping**				**Psychological distress**			
		**Coeff.**	**SE**	***p***		**Coeff.**	**SE**	***p***		**Coeff.**	**SE**	***p***
Resilience	*a*^*1*^	−.08	.01	<.001	*a*^*2*^	−.13	.03	<.001	*c’*	−.12	.07	.07
Appraisals of threat and loss		–	–	–	*d*^*21*^	1.04	.21	<.001	*b*^*1*^	3.17	.38	<.001
Passive coping		–	–	–		–	–	–	*b*^*2*^	.88	.19	<.001
Constant	*i*_*M1*_	3.49	.36	<.001	*i*_*M2*_	12.70	.89	<.001	*i*_*Y*_	3.20	2.99	.29
		*R*^*2*^ = .16				*R*^*2*^ = .35				*R*^*2*^ = .55		
		*F*(2,226) = 43.29, *p* < .001				*F*(2,225) = 50.71, *p* < .001				*F*(3,224) = 86.62, *p* < .001		

*Note. Coeff*. unstandardized regression coefficient, *SE* Standard error, *p p*-value, *n* Number of participants

**Table 5:** Total, direct and indirect effects of resilience on psychological distress (*n* = 228)

			**Effect**^**a**^	**SE**	**95% CI**
Total effect		*c*	−.55	.08	−.70, −.40
Direct effect		*c´*	−.12	.07	−.26, .01
Indirect effect	Via appraisals only	*a*^*1*^ * *b*^*1*^	−.24	.05	−.34, −.16
	Via coping only	*a*^2^* b^*2*^	−.11	.03	−.18, −.06
	Via appraisals and coping	*a*^1^* d^21^* b^*2*^	−.07	.02	−.12, −.03

*Note.* Indirect effect standard errors (SE) and confidence intervals are based on 10.000 bootstrap samples; *CI* Confidence interval, *n* Number of participants. ^a^Unstandardized regression coefficients

### Differences between subgroups (SCI and ABI)

The mediation analyses were repeated for significant others of persons with SCI versus ABI separately. All coefficients were in the same direction and absolute values were largely similar. The model of significant others of persons with SCI explained 60% of the variance in psychological distress (*F* (3,118) = 65.09, *p* < .001) and the direct relationship between resilience and psychological distress remained statistically significant after adding the mediators in the model (*c* = − 0.54, *p* < .001; *c’* = −.16, *p* < .05). In the ABI group, the model explained 47% of the variance in psychological distress (*F* (3,102) = 21.15, *p* < .001). In this group, the direct relationship between resilience and psychological distress was completely explained by indirect relationships (*c* = −.53, *p* < .001; *c’* = −.08, *p* > .05).

## Discussion

The purpose of this study was to test if psychological distress among significant others of persons with SCI or ABI in the subacute phase during first inpatient rehabilitation can be explained by the personal resource resilience, and if this relation is serially mediated by appraisals and coping. It was found that 55% of the variance in psychological distress was explained by its relationship with resilience. Furthermore, results of the serial mediation model indicate that significant others with high resilience show less psychological distress because they make less negative appraisals (threat and loss) and use less passive coping compared to significant others with low resilience. The relationship between resilience and psychological distress, and the mediation via appraisals coping, showed a similar pattern in both diagnosis groups (SCI and ABI).

### Psychological distress

The mean psychological distress score in our sample (13.1, SD = 7.8) was considerably higher than the mean score found in the general Dutch population (8.4, SD = 6.3; persons aged 18–65) [25], indicating that significant others of persons with SCI or ABI on average experience higher levels of psychological distress than persons in the general Dutch population. Respectively 40.8 and 33.8% of the significant others reported high symptoms of anxiety or depression (45.9 and 39.3% in the SCI group; 34.9 and 27.4% in the ABI group). A literature review focusing on caregivers of persons with stroke showed that 21.4% had anxiety symptoms and 40.2% depressive symptoms [7]. In our study, symptoms of anxiety were more common than symptoms of depression, while in the review the opposite was found. Probably this difference could be explained by differences in the time of assessment. There are indications that, in contrast to levels of depression, levels of anxiety are higher in the subacute phase and decline over time [21]. This may explain the higher percentages of significant others reporting symptoms of anxiety in the current study (in the subacute phase) and the lower percentages found in the review (mostly in the chronic phase) [7].

### Mediation model

This is the first study focusing on psychological determinants of psychological distress among significant others of persons with SCI or ABI using a serial multiple mediation model. First of all, correlations between resilience, appraisals of threat and loss, passive coping, and psychological distress were all moderate to strong and in the expected direction based on the stress-coping theory [10], and previous research findings among significant others [16–18, 21, 30]. Furthermore, we found support for the mediating effect of appraisals of threat and loss, and passive coping in the explanation of psychological distress among significant others, as was previously found among caregivers of patients with cancer, and which is in line with results found among persons with SCI [11, 12]. This seems to support the idea that the adaptation process of significant others of persons with SCI or ABI is essentially the same as that of significant others in other diagnosis groups and patients. This suggest the general applicability of the stress-coping modal as a behavioral model.

Based on the stress-coping model, health-related factors of patients could be considered as a (extra) stress factor [10]. Therefore, it seems noteworthy that in our study the level of physical independence of the person with SCI or ABI was not found to be related with psychological distress, resilience, appraisals and coping. However, also in previous studies no strong relationships were found between physical independence of the patient and anxiety, depression or mental health of caregivers [21, 43]. This could indicates that the objective severity of disabilities is subordinate to the subjective experience of the situation [43].

In the ABI group, the direct relationship between resilience and psychological distress disappeared after adding the indirect relationships via appraisals of threat and loss, and passive coping in the model, while this direct relationship remained significant after adding the mediators in the model in the SCI group. However, also in the SCI subgroup the main part of the relationship between resilience and psychological distress was explained by mediation. The regression coefficients in both subgroups were all in the same direction and differences in absolute values between the models in the ABI and SCI groups were small. So, overall, we conclude that the mediation model is similar in both subgroups. Because this is the first study in which the applicability of the theoretical stress-coping model is tested among significant others of persons in different diagnosis groups, we were not able to compare our results with previous results.

### Implications

To be able to support significant others to handle psychological distress early after onset of injury, it is important to understand the underlying mechanism. The present study indicates that resilience, appraisals of threat and loss, and passive coping are psychological factors that should be taken into account. Based on these findings it seems useful to examine the changeability of resilience, appraisals and coping and to investigate the effectiveness of interventions focusing on these psychological factors. In the prevention or reduction of psychological distress, interventions could aim to increase resilience, to reduce negative appraisals and to deploy less passive coping strategies in problematic situations. Programs for counseling family members that have been developed and are being applied in recent years for carers in other diagnosis groups, mainly consist of psychoeducation, using techniques focusing on problem-solving, self-management, coping with the new situation and stress reduction [44]. Such interventions seem to fit well with our findings. Among carers of persons with dementia evidence was found that psychoeducational programs based on the Cognitive Behavioral Theory or Acceptance and Commitment Therapy seemed beneficial for treating psychological distress [45]. However, more controlled studies on the application of these programs during the transitions from hospital or rehabilitation center to home are needed before clear recommendations to healthcare professionals can be made regarding optimal time, format, dosage, and characteristics of the target population of programs to support caregivers of persons with SCI and ABI [44].

### Strengths and limitations

Unique to the present study is that we measured outcomes among a large group of significant others shortly after admittance of the person with SCI or ABI to first inpatient rehabilitation in one of the twelve participating rehabilitation centers spread across the Netherlands. Furthermore, testing a serial multiple mediation model based on the stress-coping model of Lazarus and Folkman [10] in a sample of significant others of persons with SCI or ABI is new. Our study has some limitations. First, this study concerned a selective group of significant others of persons with SCI or ABI who were admitted to rehabilitation facilities. Significant others of persons who were discharged home or to a nursing home after their stay in the hospital were not included. Second, results should be interpreted cautiously given the cross-sectional design of the study which makes it impossible to make any statements about causality or seriality. Our interpretations of the findings are based on the theoretical assumptions of the stress-coping model. A longitudinal study is needed to confirm our findings over time. Third, there are several personal resource factors that could be relevant. However, only one independent variable could be added in the mediation model. We have chosen to include resilience because previous research had demonstrated that resilience is a strong predictor of psychological distress [16–18]. We realize that we are not yet aware of the possible role of other factors such as self-efficacy [21]. Fourth, besides demographic variables, we only included physical independence and diagnosis of the person with SCI or ABI as potential confounders. For instance, we did not include cognition in the analyses, because we only got information about cognition for the group of persons with ABI (not for SCI). However, especially in the ABI group, cognition may be a stress factor, that may be more of a burden for the significant other over time. We decided not to include cognition in the ABI model, in order to keep the models for SCI and ABI comparable. Fifth, we did not include health-related factors of the significant others and factors representing their social and physical context in our model, while health-related factors and social and physical context are part of the stress-coping model of Lazarus and Folkman [10]. Previous research showed that, for instance, social support relates with resilience [46], appraisals [47], coping [48], and psychological distress [21, 49]. So in further investigation of the theoretical model, it is recommended to take these factors into account. Last, we have used the stress-coping model as theoretical framework in the explanation of psychological distress. There may also be other factors of interest in the explanation of distress that do not feature in this theoretical model and which we have not assessed. However, the mediation model tested in the present study already explained a relative large part of the variance in psychological distress (55%).

## Conclusions

Psychological distress is common among significant others of persons with SCI and ABI. Resilience, appraisals of threat and loss, and passive coping seem to be important psychological factors in the explanation of psychological distress. Therefore, it seems useful to investigate if such psychological factors are changeable and if intervention programs which focus on these factors are effective in order to prevent or reduce psychological distress among significant others.

## Data Availability

The datasets used and analysed during the current study are available from the corresponding author on reasonable request.

## Abbreviations

AIS: American Spinal Injury Association Impairment Scale
ASIA: American Spinal Injury Association
ABI: Acquired brain injury
ALE: Appraisals of Life Events
CD-RISC-10: Connor-Davidson Resilience Scale
HADS: Hospital Anxiety and Depression Scale
SCI: Spinal cord injury
UCL: Utrecht Coping List
USER: Utrecht Scale for Evaluation of Rehabilitation
VIF: Variance inflation factors
